# Efficacy of Hydroxychloroquine for Improving Pregnancy Outcomes in a Female with Systemic Lupus Erythematosus and Antiphospholipid Syndrome

**DOI:** 10.1155/2022/5612091

**Published:** 2022-08-21

**Authors:** Yumika Takaki, Atsushi Daimon, Misa Nunode, Tomohito Tanaka, Daisuke Fujita, Masahide Ohmichi

**Affiliations:** Department of Obstetrics and Gynecology, Osaka Medical and Pharmaceutical University, 2-7 Daigaku-Machi, Takatsuki, Osaka 569-8686, Japan

## Abstract

The use of heparin and low-dose aspirin is the current conventional treatment for pregnant females with antiphospholipid syndrome (APS). However, there is no additional treatment recommended for cases where the standard treatment cannot prevent obstetric complications such as fetal loss and placental insufficiency. Recently, the addition of a novel antimalarial, hydroxychloroquine (HCQ), to the conventional treatment has shown the potential to prevent obstetric complications. Herein, we report a case in which the addition of HCQ improved adverse pregnancy outcomes in a pregnant female with systemic lupus erythematosus and aspirin-heparin-resistant APS.

## 1. Introduction

Females with antiphospholipid syndrome (APS) have an increased risk of thrombosis and pregnancy complications, such as recurrent early miscarriages and hypertensive disorders of pregnancy [1]. The use of heparin and low-dose aspirin (LDA) is the current conventional treatment for pregnant females with APS and has improved pregnancy outcomes to a live birth rate of >70% [2, 3]. Recently, an antimalarial drug, hydroxychloroquine (HCQ), was recommended, besides the conventional treatment in patients with APS and previous pregnancy failure on current therapy for APS [4]. Herein, we report a case of systemic lupus erythematosus (SLE) and aspirin-heparin-resistant APS successfully treated with HCQ in addition to the patient's existing therapy.

## 2. Case Presentation

**Figure 1:**
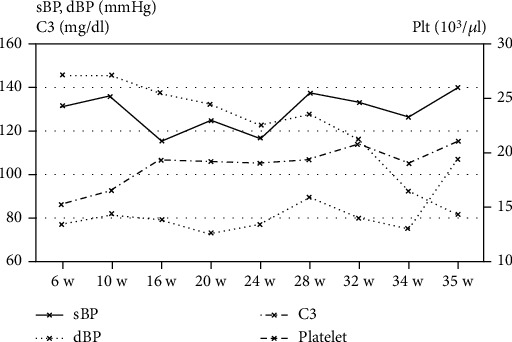
Changes in blood pressure and blood sampling data.

A 35-year-old pregnant female (gravida 4, parity 1) with SLE without lupus nephritis and APS visited our hospital at 6 weeks of gestation (GW). The patient was diagnosed with SLE without lupus nephritis and was positive for antiphospholipid antibody (aPL), including lupus anticoagulant, anticardiolipin antibody, and antiphosphatidylserine/prothrombin antibodies at 18 years old. The patient's pregnancy history is presented in Table 1. The first pregnancy resulted in an APS diagnosis. The SLE remained in remission, and this case was diagnosed as an aspirin-heparin-resistant APS based on the patient's pregnancy history. Consequently, for the fourth pregnancy, HCQ was commenced before conception with the patient's informed consent. Immediately after a positive pregnancy test was obtained, warfarin was discontinued. Following that, the 6 GW embryo was confirmed, therapeutic doses of unfractionated heparin (UFH) (15,000 units/day, subcutaneously) were introduced, and intravenous immunoglobulin therapy (IVIg) (25 g/day for 5 consecutive days; total dose: 125 g) was administered without adverse effects. LDA was maintained until 34 GW, and the fetal growth was appropriate for the gestational age. However, the patient's platelet count decreased from 27.2 × 10^4/^*μ*L at 6 GW to 16.5 × 10^4/^*μ*L at 34 GW, and the maternal blood pressure was elevated to 133/90 mmHg (Figure 1). Contrarily, the serum C3 levels did not decrease during this pregnancy (Figure 1). The platelet count further decreased to 14.4 × 10^4/^*μ*L at 35 GW (Figure 1), and we assessed that the patient's condition had worsened. Therefore, a cesarean section was performed, and a male infant weighing 2,618 g was delivered. Continuous heparin infusion was initiated 12 h after cesarean section and continued for 5 days. On postpartum day 1, LDA (100 mg/day) and warfarin (5 mg/day) were restarted. The patient and infant were discharged without complications on postpartum day 6.

## 3. Discussion

This case of treatment-resistant APS with SLE was treated successfully with HCQ, LDA, therapeutic doses of UFH, prednisolone (PSL), tacrolimus, and IVIg at 6 GW.

Not only autoimmune diseases, such as SLE and APS [1], but also other proinflammatory diseases, such as endometriosis [5] and polycystic ovary syndrome [6], are known to influence the reproductive outcomes. Despite receiving conventional therapy, 20–30% of females with APS continue to experience pregnancy complications. Attempts have been made to add PSL [7] or IVIg [8] to the conventional treatment; however, the best approach to improve the outcomes of these pregnancies is unknown. Recently, novel drugs, including HCQ [9] and pravastatin [10], have been considered for preventing adverse pregnancy outcomes. HCQ was recommended in addition to the conventional therapy in patients with APS and previous pregnancy failure receiving the standard treatment [4]. Thus, HCQ may benefit patients with thrombotic and obstetric APS.

Several studies have investigated the mechanism by which HCQ exerts an antithrombotic effect. In vitro, HCQ inhibited platelet aggregation and the release of arachidonic acid from aPL-induced stimulated platelets [11]. In vivo, in patients with aPL and APS, HCQ restored the destruction of the anticoagulant annexin A5 [12] and decreased soluble tissue factor [13]. The obstetric effects of HCQ have been reported in experiments with mice. In an aPL-induced fetal loss mouse model, HCQ prevented fetal death, increased placental and fetal weight, and decreased placental superoxide production, which is a marker of oxidative stress [14]. In vitro, HCQ significantly reduced the induction of endosomal nicotinamide adenine dinucleotide phosphate (NADPH), an enzyme complex involved in proinflammatory signaling pathways [15]. Clinically, HCQ is expected to prevent aPL-related adverse pregnancy outcomes, including first-trimester miscarriages and ischemic placental-mediated complications, such as preeclampsia and fetal growth restriction [9, 16].

In conclusion, HCQ may be useful in improving pregnancy outcomes in patients with previous pregnancy failure receiving the current conventional treatment for APS.

## Figures and Tables

**Table 1:** Patient's pregnancy history.

	First pregnancy	Second pregnancy	Third pregnancy
Age (y)	28	31	34
Medication during pregnancy	PSL (9 mg/day)	PSL (15 mg/day)	PSL (12 mg/day)
	LDA (100 mg/day)	LDA (100 mg/day)	LDA (100 mg/day)
		UFH (10,000 units/day)	UFH (10,000 units/day)
			TAC (4 mg/day)
			IVIg at 9 GW (25 g/day, 5 consecutive days, total 125 g)
Complication: mother	None	HELLP syndrome	Chronic hypertension
Complication: infant	FGR, IUFD	Intact survival	IUFD
Gestational age at delivery (GW)	22	24	16
Birth weight (g)	145	498	80

PSL: prednisolone; LDA: low-dose aspirin; UFH: unfractionated heparin; TAC: tacrolimus; FGR: fetal growth restriction; IUFD: intrauterine fetal demise; HELLP: hemolysis, elevated liver enzymes, and low platelets; GW: gestational week; IVIg: intravenous immunoglobulin.

## Data Availability

Data is available upon request by emailing atsushi.daimon@ompu.ac.jp.
